# Deregulated MicroRNA-21 Expression in Monocytes from HIV-Infected Patients Contributes to Elevated IP-10 Secretion in HIV Infection

**DOI:** 10.3389/fimmu.2017.01122

**Published:** 2017-09-11

**Authors:** Xian Wu, Le-Le Zhang, Lin-Bo Yin, Ya-Jing Fu, Yong-Jun Jiang, Hai-Bo Ding, Zhen-Xing Chu, Hong Shang, Zi-Ning Zhang

**Affiliations:** ^1^Key Laboratory of AIDS Immunology of National Health and Family Planning Commission, Department of Laboratory Medicine, The First Affiliated Hospital, China Medical University, Shenyang, China; ^2^Collaborative Innovation Center for Diagnosis and Treatment of Infectious Diseases, Hangzhou, China

**Keywords:** interferon-inducible protein 10, microRNA-21, IFN-stimulated gene 15, inflammatory response, HIV infection

## Abstract

Persistent activation and inflammation impair immune response and trigger disease progression in HIV infection. Emerging evidence supports the supposition that excessive production of interferon-inducible protein 10 (IP-10), a critical inflammatory cytokine, leads to immune dysfunction and disease progression in HIV infection. In this study, we sought to elucidate the cause of the upregulated production of IP-10 in HIV infection and explore the underlying mechanisms. Bolstering miR-21 levels using mimics resulted in the obvious suppression of lipopolysaccharide (LPS)-induced IP-10 in monocyte leukemia cells THP-1 and vice versa. The analysis of the primary monocytes of HIV patients revealed significantly less miR-21 than in healthy controls; this was opposite to the tendency of IP-10 levels in plasma. The secretion of IP-10 due to LPS stimulation was not affected by miR-21 modulation in the differentiated THP-1 macrophages (THP-1-MA). We found a novel switch, IFN-stimulated gene 15 (*ISG15*), which triggers the expression of IP-10 and is significantly upregulated during the differentiation of THP-1 into THP-1-MA. The inhibition of ISG15 can restore the regulation of IP-10 by miR-21. In summary, IP-10 expression in monocytes is regulated by miR-21, whereas in macrophages, this fine-tuning is attenuated by the enhanced expression of ISG15. This study paves the way to a comprehensive understanding of the molecular regulatory mechanism of IP-10, a key point in immune intervention strategy.

## Introduction

The deterioration of the gut epithelium and enlarged microbial translocation triggered by the rapid depletion of gastrointestinal CD4^+^ T cells leads to a persistent, systemic activation, and inflammation of HIV infections (1, 2). Antiretroviral therapy (ART) extends the lifespan and the quality of life of HIV-infected patients while completely eliminating chronic immune activation and inflammation does not occur (3, 4). Persistent inflammation contributes to immune senescence and exhaustion, factors linked to most non-AIDS-related complications of HIV, such as cardiovascular, neurocognitive, and metabolic syndromes (2–5). A comprehensive understanding of the molecular and cellular basis of the inflammatory status allows these remaining challenges of HIV treatment to be addressed.

Excessive chronic production of inflammatory cytokines, including TNF-α, IFN-γ, interleukin (IL)-18, and interferon-inducible protein 10 (IP-10), has been recorded in HIV-infected subjects (6–8); among these, IP-10 is functionally categorized as an inflammatory chemokine which attributed to inflammatory disorders, immune dysfunction, and tumor development (9). In HIV infection, elevated blood levels of IP-10 are associated with rapid disease progression and persistent immune activation (6, 10–12). Previous studies found that IP-10 decreased the function of T cells (13) and NK cells (14) and stimulated HIV replication (15). This indicates that the regulation of IP-10 expression in HIV infection influences the delay of inflammation, slowing down disease progression. Studies have shown that HIV-1 infection induces the production of IP-10 through the TLR7/9 dependent (16) or MDA5/Rig-I pathways (17). However, the factors triggering the significant upregulation of IP-10, especially at a post-transcriptional level in HIV infection have not been clearly elucidated.

The correlation between microRNAs (miRNAs), a species of small RNA molecules that are important in gene regulation, and HIV-1 infection emerged as a promising platform to understand the complex molecular mechanisms that occur in infection (18). More and more evidence supports the contention that the changes within the endogenous miRNA levels observed after HIV infection are essential to controlling HIV-1 replication and latency (19) and modulating the key targets associated with T cell exhaustion (20). Several previous studies have shown multiple processes of cross-regulation between cytokine and the miRNA pathways (21). In addition, miRNA influences immune function by inducing changes in cytokine, such as interleukin-2 (IL-2) and IL-10 in HIV infection (22, 23). The potential of miRNA to regulate IP-10 in HIV infection has not yet been reported.

In this study, we investigated miRNAs that might target IP-10 and suppress its level in monocytes. We found that miR-21 could target and suppress the secretion of IP-10 in monocytes and that HIV-infected patients exhibited a decreased level of miR-21 in monocytes. However, the modulation of the expression of miR-21 had no effect on IP-10 in macrophages, which is partly ascribed to the elevated expression levels of ISG15 in these cells. The data showed that miR-21 regulated levels of IP-10, which paves the way to a comprehensive understanding of the molecular regulatory mechanism of IP-10.

## Materials and Methods

### Patient Selection

In total, 32 treatment-naive HIV-infected patients and 35 healthy controls (HCs) participated in this study. The demographic information and clinical characteristics of the subjects are listed in Table 1. Peripheral blood mononuclear cells (PBMCs) were freshly isolated by Ficoll centrifugation, and plasma samples were collected. Ethical approval was obtained from the First Hospital of China Medical University, and all participants were informed of the collection of blood samples and provided written consent prior to enrolment in the study.

**Table 1:** Demographic and clinical characteristics of subjects.

Characteristic	HIV infected patients	Normal controls
Subject no.	32	35
Age (years, mean ± SD)	34 ± 6	36 ± 6
Male (No, %)	15 (46.9%)	15 (42.9%)
Han Ethnic (No, %)	32 (100%)	35 (100%)
CD4 (cells/μL, mean ± SD)	331 ± 235	N/A
VL [log copies/mL (mean ± SD)]	4.15 ± 1.09	N/A
Time since diagnosis (weeks, mean, range)	65 (1–336)	N/A

### Primary Cells Isolation and THP-1 Cell Culture

For primary cell isolation, monocytes (CD3^−^CD14^+^), NK cells (CD3^−^CD56^+^CD16^+^), CD4^+^ T cells (CD3^+^CD4^+^), CD8^+^ T cells (CD3^+^CD8^+^), and B cells (CD3^−^CD19^+^) were selected from PBMCs using a BD FACS Aria flow cytometer. THP-1 human acute monocyte leukemia cells were cultured in RPMI 1640 supplemented with 10% FBS (HyClone). To promote the differentiation of THP-1 to THP-1 macrophages (THP-1-MA) cells, the THP-1 were treated with phorbol 12-myristate 13-acetate (PMA; 100 ng/mL; Sigma) for 48 h (24).

### miRNA Mimics, Inhibitors, and siRNA Delivery

The transfection of miRNA mimics, inhibitors, and controls (GenePharma) were performed with HiperFect Transfection Reagent (Qiagen). Briefly, 20 µM mimics or inhibitors were transfected into THP-1, THP-1-MA cells for 48 h. Subsequently, the THP-1 cells were stimulated using lipopolysaccharide (LPS; 1 µg/mL; Sigma), and incubated for a further 24 h. The cells were collected for RNA extraction, and the supernatants were collected for IP-10 detection. Transfection of primary CD14^+^ monocytes with mimics was performed with RNAiMAX (Invitrogen) according to the manufacturer’s protocol. The sequences of the mimics and inhibitors are listed in Table S1 in Supplementary Material. The forced reduction process of ISG15 was achieved by employing the 20 µM ISG15 siRNA for 24 h (Invitrogen). This was followed by THP-1 cell treatment with PMA for 48 h, into which was transfected 20 nM mimics by HiperFect Transfection Reagent (Qiagen). For the inhibition of ISG15 in THP-1 cells, IG15 siRNA was transfected to THP-1 cells for 24 h. The siRNA control was represented by non-specific Stealth RNAi^®^ Negative Control Duplexes.

### Reverse Transcription and Quantitative Real-time PCR

MicroRNA was extracted from cells using the miRNeasy Micro kit (Qiagen). Total RNA was isolated using the RNeasy Micro kit (Qiagen), and the purified RNA was treated with DNase I reagent to eliminate genomic DNA contamination. The RNA was reversely transcribed using Primpscript^®^RT reagent kit (TAKARA) according to the instructions provided by the manufacturer. The real-time PCRs for the detection of miRNA and mRNA were performed with SYBR^®^ Premix Ex Taq™ II (TAKARA). All the primer sequences are listed in Table S1 in Supplementary Material. The levels of miRNA expression were normalized to small nucleolar RNA (snRU6), while mRNA expression was normalized to GAPDH. The relative expression levels of miRNA and mRNA were calculated based on the change in cycling threshold method as 2^−ΔΔCt^.

### IP-10 Detection

The measurements of supernatant IP-10 produced from cell culture and plasma from HIV-infected patients and HCs were performed using an ELISA kit (R&D Systems). Concentrations of IP-10 were calculated using the standard curve and multiplied by the dilution factor.

### Luciferase Activity Assay

According to the target prediction, the IP-10 3′UTR contained two appropriate sequences with miR-21 binding sites (196–202 and 235–240 nt). The 3′UTR fragments (synthesized by GenePharma), containing the putative binding sites for miR-21, were cloned into the GP-miRGLO Vector. The luciferase reporter assay was performed in 293T cells cultured in 48-well plates. The 293T cells were co-transfected with the IP-10 3′UTR reporter (wild type, mut-1, mut-2), empty vector plasmid (GP-miRGLO), and appropriate miRNA mimics and inhibitors (20 µM), using the Lipofectamine 2000 reagent (Invitrogen). At 24 h after transfection, firefly and renilla luciferase activities were measured using the Dual-Luciferase Report Assay (Promega).

### Statistical Analysis

GraphPad Prism was used to conduct statistical analyses, and the comparison between the miRNA and IP-10 levels was performed using the Student’s *t*-test. The non-parametric Mann–Whitney test was used to determine the differences between HIV patients and HCs. The correlations between variables were evaluated using the Spearman’s correlation coefficient, and all *P* values under 0.05 were considered statistically significant.

## Results

### Elevated IP-10 Levels in HIV-Infected Patients Are Linked to Disease Progression

**Figure 1:**
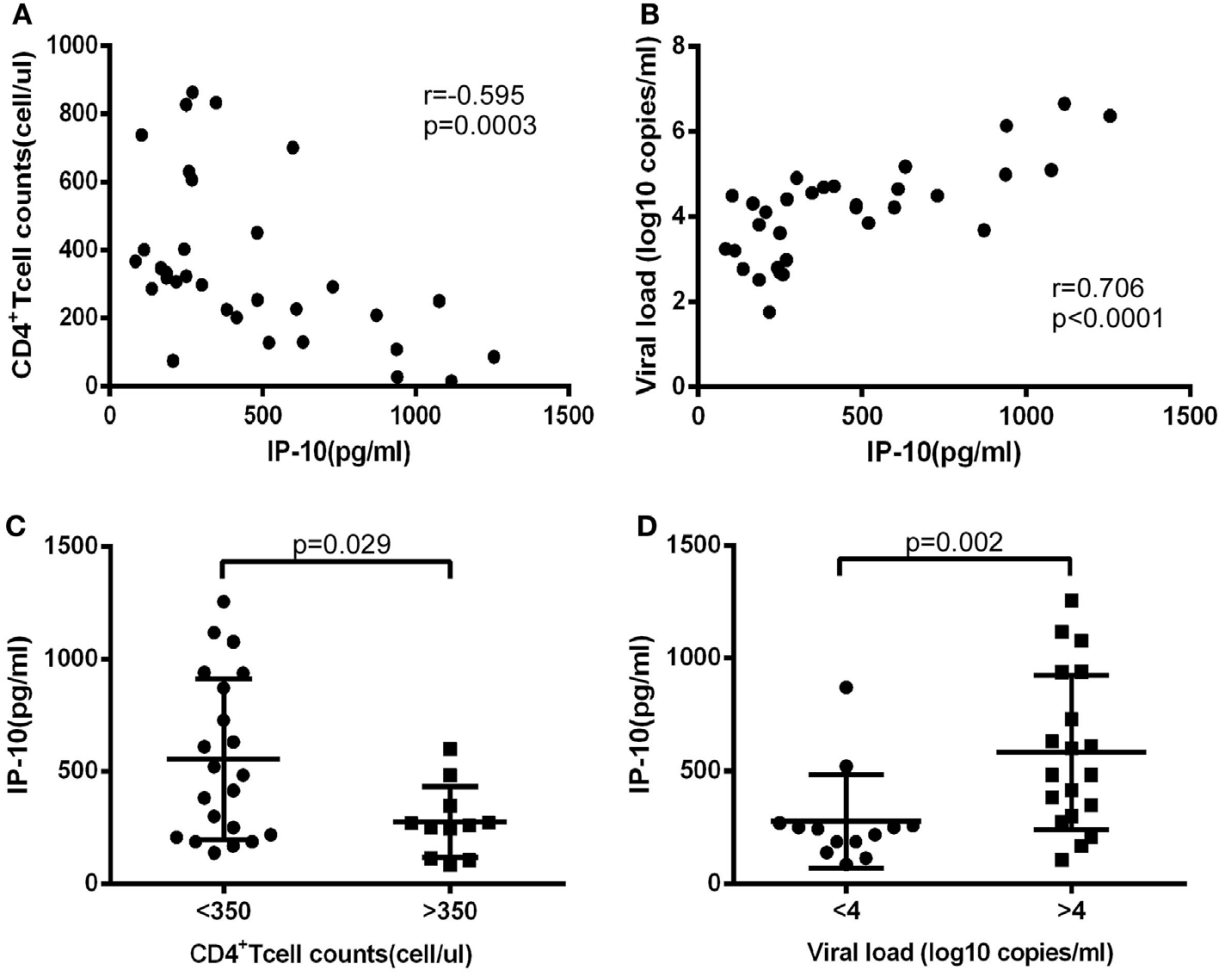
The increased interferon-inducible protein 10 (IP-10) level was association with progression of HIV infection. The relationship between plasma IP-10 and CD4^+^ T cell counts **(A)**, viral load **(B)** in HIV-infected patients; **(C)** comparison of plasma IP-10 in HIV-infected patients in high (>350 cells/μL) and low (<350 cells/μL) CD4^+^ T cell count groups; **(D)** comparison of plasma IP-10 in HIV-infected patients in low (<4 log copies/mL) and high (>4 log copies/mL) viral load groups.

First, we investigated levels of IP-10 secretion in the HIV-infected patients enrolled in our study. All of these patients had considerably greater IP-10 plasma concentrations (458.0 ± 329.5 pg/mL) than HCs (115.0 ± 56.3 pg/mL) (*P* < 0.0001; Figure S1 in Supplementary Material). Then we studied the association of levels of IP-10 with disease progression. We found that IP-10 was negatively correlated with CD4^+^ T-cell counts (*r* = −0.595, *P* = 0.0003; Figure 1A) and positively correlated with viral load (*r* = 0.706, *P* < 0.0001; Figure 1B). We then divided the patients into two groups according to CD4^+^ T cell counts and viral load. IP-10 levels were significantly higher in the group of patients with seriously reduced CD4^+^ T cell counts (<350 cells/μL; *P* = 0.029; Figure 1C) and the group with high viral loads (LogVL > 4; *P* = 0.002; Figure 1D). The correlation between IP-10 and disease progression confirmed that IP-10 is a good biomarker in HIV disease progression.

### Identification of Individual miRNAs for IP-10 Production Regulation

**Figure 2:**
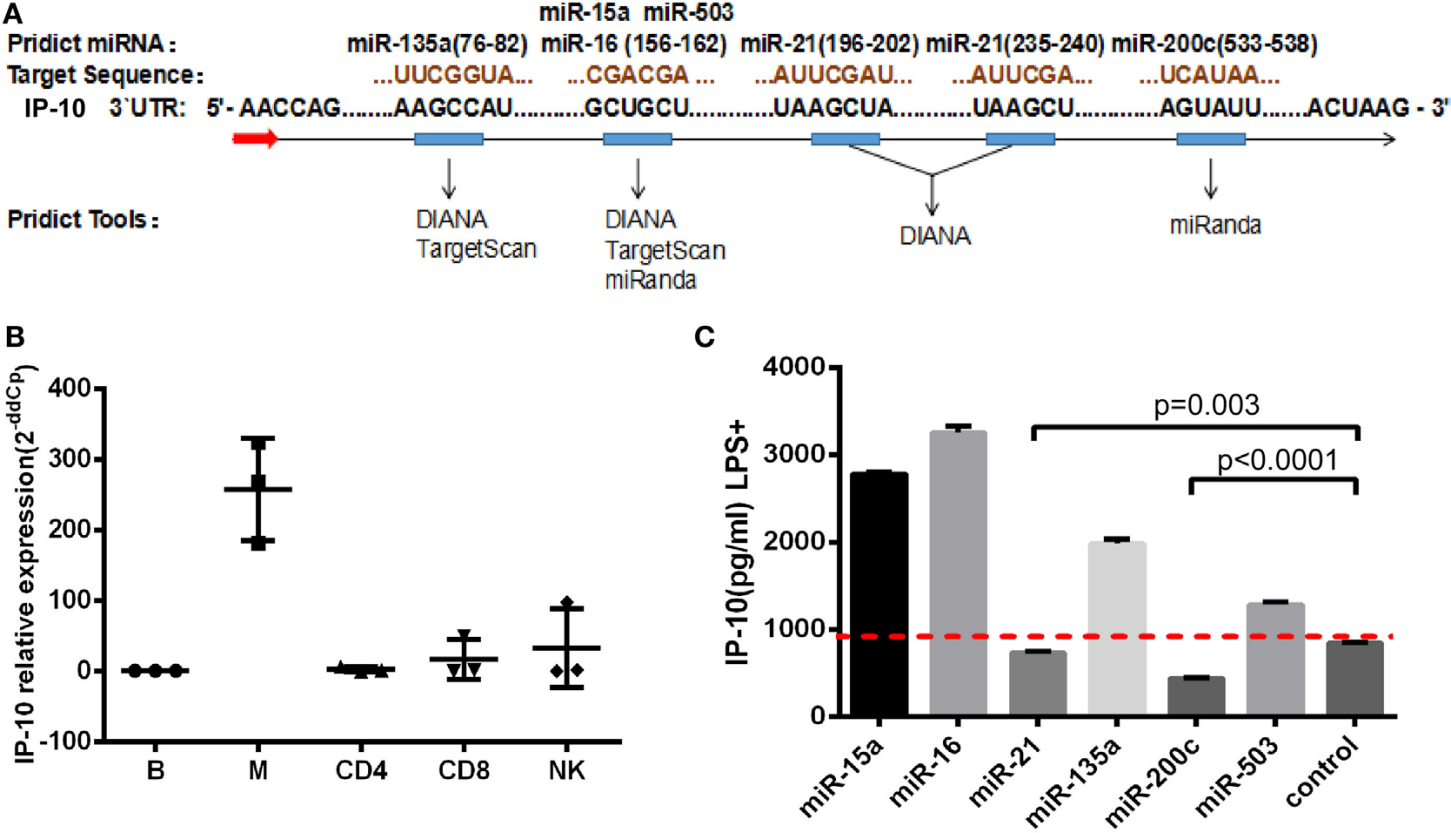
miR-21 identified as potential regulator of interferon-inducible protein 10 (IP-10) secretion. miRNA candidates were screened, using three target-prediction software programs, including TargetScan, miRanda, and DIANA for binding sites. **(A)** miRNAs and IP-10 3′UTR matching sequences; **(B)** IP-10 mRNA expression exhibited by B cells, monocytes, CD4^+^ and CD8^+^ T cells, and NK cells; **(C)** THP-1 cells were transfected with either mimics of six miRNAs or negative control for 48 h and stimulated by lipopolysaccharide (LPS) for 24 h. IP-10 production in supernatants of THP-1 cells was measured.

Although the key role played by IP-10 in HIV infection has been recognized, the factors regulating its expression, particularly at post-transcriptional level, have not been investigated. Given that miRNAs act as repressors and have the propensity to fine-tune gene expression, we sought to explore whether miRNAs can regulate IP-10 expression. Three miRNA target prediction tools were employed to identify miRNA candidates: TargetScan, miRanda, and DIANA. These online tools showed that six miRNAs possessed a conserved 6–7 mer seed matching the IP-10 3′UTR. These were miR-15a, miR-16, miR-21, miR-135a, miR-200c, and miR-503. As illustrated in Figure 2A, IP-10 exhibited two binding sites for miR-21 and just one for the other miRNAs.

Early HIV-infected patients, whose durations of infection were 0–40 weeks, display considerably distinct IP-10 mRNA levels among T cells, mDCs, monocytes, and B-cells, according to previously reported studies (16). Thus, we initially investigated the variation of IP-10 mRNA levels of expression among a series of cells, sorted B-cells, monocytes, NK, CD4^+^, and CD8^+^ T cells supplied by healthy donors, and found that monocytes had the highest levels of expression of IP-10 mRNA (Figure 2B), which is consistent with previous studies. Therefore, THP-1 cells, a human acute monocyte leukemia cell line, were selected to perform the *in vitro* experiments. The THP-1 cells were overexpressed for the six miRNA candidates and stimulated with LPS (25) to identify the miRNAs which could regulate IP-10 production. IP-10 secretion was largely reduced by the overexpression of miR-21 (*P* = 0.003) and miR-200c (*P* < 0.0001) compared to the control (Figure 2C). Because the overexpression multiples of miR-200c were too high (data not shown) to be achieved *in vivo*, only miR-21 was identified for further studies.

### miR-21 Regulates IP-10 Secretion through THP-1 Cells

**Figure 3:**
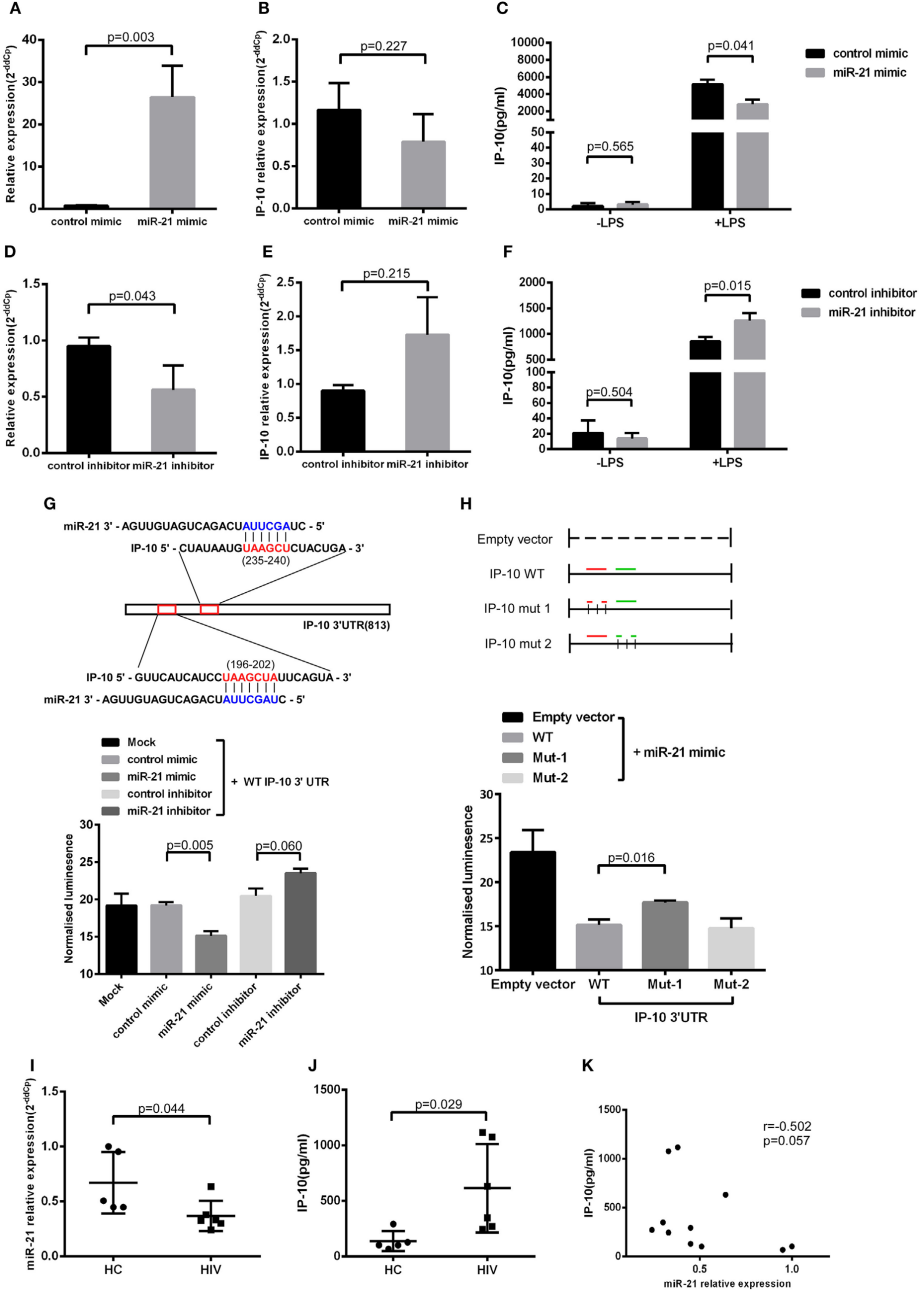
miR-21 regulates interferon-inducible protein 10 (IP-10) secretion in THP-1 cells. THP-1 cells were transfected with either miR-21 mimics or control for 48 h and subsequently stimulated by lipopolysaccharide (LPS) for another 24 h. **(A)** The transfection of miR-21 mimics led to enhanced expression of miR-21 in THP-1 cells. Following the transfection, IP-10 mRNA expression **(B)** and secretion after LPS stimulation **(C)** were measured. THP-1 cells were transfected with either miR-21 inhibitors or control over 48 h and stimulated by LPS for 24 h. **(D)** The transfection of miR-21 inhibitors triggered a decreased expression of miR-21 in THP-1 cells. The inhibition of miR-21 did not influence IP-10 mRNA levels **(E)** yet did increase IP-10 secretion **(F)**. **(G)** Two conserved miR-21 binding sites located at the IP-10 3′UTR. Luciferase activity was significantly lower in mimic-treated cells but was slightly higher in fluorescence intensity of inhibitor-treatment cells than in the mock cells. **(H)** Co-transfected with miR-21 mimic and IP-10 3′UTR mut-2 (235–240), not mut-1 (196–202) vector showed more downregulated luciferase expression than the empty vector control. **(I)** Comparison of miR-21 expression in sorted monocytes from HIV and healthy controls (HCs). **(J)** Comparison of plasma IP-10 levels in HIV and HC. **(K)** Correlation between the miR-21 expression in CD14^+^ monocytes and plasma IP-10 level.

Next, we transfected miR-21 mimics to the THP-1 cells (Figure 3A), finding that miR-21 overexpression had no effect on IP-10 mRNA levels (Figure 3B). Distinctively, the transfection of mimics greatly inhibited the IP-10 protein released from LPS-stimulated THP-1 cells (*P* = 0.041; Figure 3C), indicated that miR-21 directly targeting IP-10 at the post-transcriptional level. IP-10 as an inflammatory cytokine is almost undetectable within the supernatant of THP-1 cells, and its secretion can be stimulated by LPS.

To further evaluate the relationship between miR-21 and IP-10, miR-21 inhibitors were transfected to THP-1 cells (Figure 3D). In line with the abovementioned trends, miR-21 inhibitors did not significantly increase the IP-10 mRNA expression but efficiently enhanced IP-10 production in THP-1 cells after LPS stimulation (*P* = 0.015; Figures 3E,F). The miRNA inhibited the level of the target gene through two main mechanisms, including mRNA degradation and the blockade of translation at the steps of initiation or elongation (26). Our results suggest that miR-21 altered the protein, but not mRNA expression level of IP-10.

### Identification of IP-10 As Direct Target of miR-21

We explored the potential of miR-21 to directly target the 3′UTR of IP-10. The two putative binding sites between IP-10 3′UTR and miR-21 are shown in Figure 3G. Luciferase report vectors carrying the full 3′UTR of wild-type (WT) IP-10, mutation site 1 (mut-1, 196–202), or mutation site 2 (mut-2, 235–240) were constructed. Reporters were transfected into 293T cells with either miR-21 mimics or inhibitors, and luminescence in miR-21 mimic-treated cells was clearly less than in controls (*P* = 0.005). Furthermore, increasing fluorescent properties were ascribed to the suppression of endogenous miR-21, a phenomenon not observed in the controls (*P* = 0.060; Figure 3G). The results show that miR-21 can directly target the 3′UTR of IP-10 and affect subsequent transcription. Therefore, we also worked to identify the effective binding site between IP-10 and miR-21. No substantial differences in the inhibition luciferase activity between the WT reporter and mut-2 were observed, and transfection with mut-1 reporter had no impact on luciferase activity (Figure 3H). According to our results, miR-21 can directly target IP-10 3′UTR through bind site 1 (196–202).

### miR-21 Is Downregulated in Monocyte of HIV-Infected Patients

We have demonstrated the miR-21 potential to regulate IP-10 secretion. Further studies on the association of increased plasma levels of IP-10 observed in HIV-infected patients with miR-21 alteration in primary monocytes were performed. Because monocyte is a main source of IP-10 (16), we sorted monocytes from HIV-infected individuals (*n* = 6) as well as HCs (*n* = 5) to detect the relative expression of miR-21. The levels of miR-21 in monocytes of HIV-infected patients were considerably lower (*P* = 0.044; Figure 3I) than those of HCs, whereas IP-10 levels in plasma exhibited the opposite tendency (*P* = 0.029, Figure 3J). There was a trend of negative correlation between miR-21 expression in monocytes and plasma IP-10 concentrations (*r* = −0.502, *P* = 0.057; Figure 3K). We found that overexpression of miR-21 in primary monocytes (*P* = 0.002; Figure S2A in Supplementary Material) from HIV-infected patients has the decreasing tendency of IP-10 production by LPS stimulation (*P* = 0.057; Figure S2B in Supplementary Material). The results suggesting that miR-21 may contribute to the regulation of IP-10 production in monocytes in the context of HIV infection.

### Elevated Expression of ISG15 Weakens the Regulation of IP-10 by miR-21 in THP-1-MA Cells

**Figure 4:**
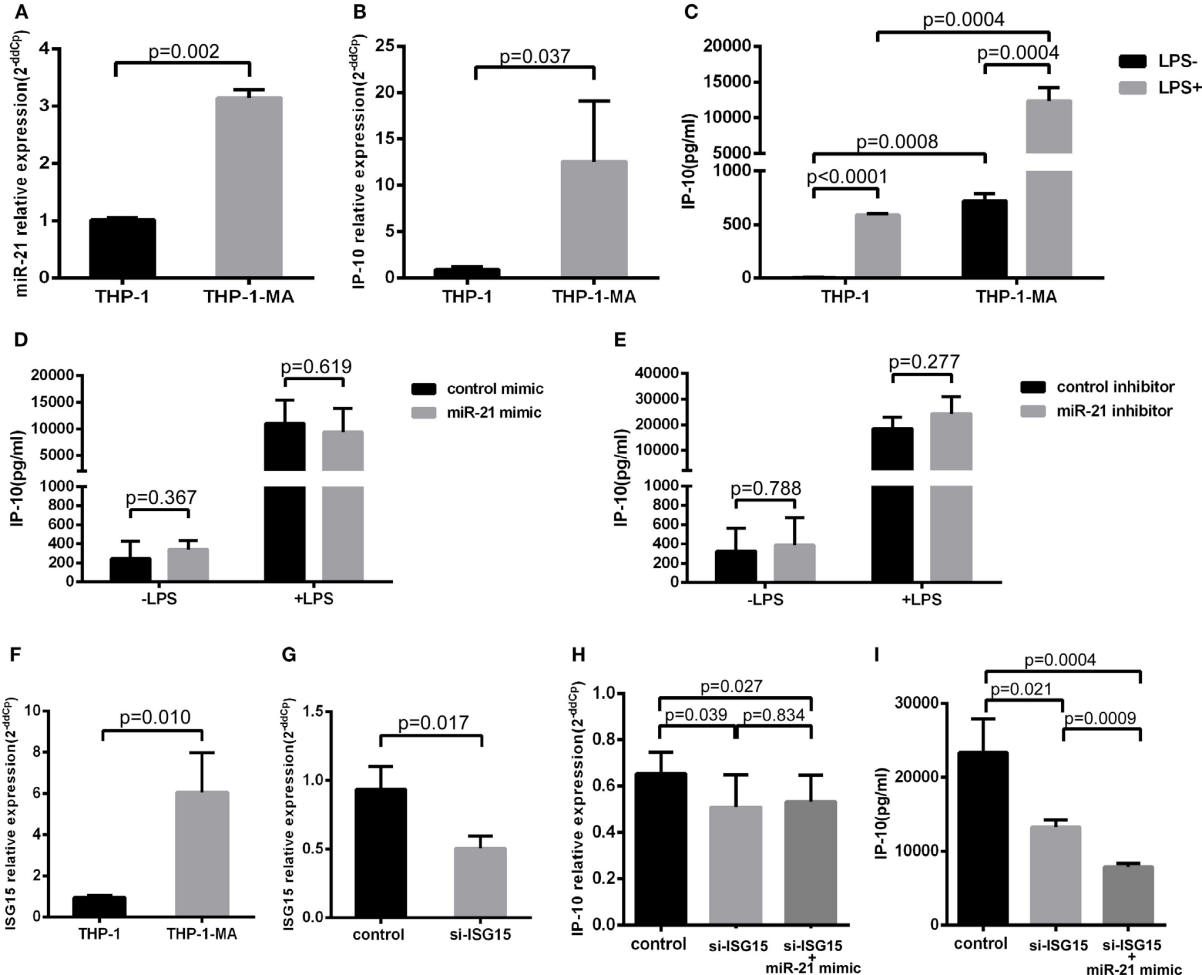
IFN-stimulated gene 15 (ISG15) weakens the function of miR-21 regulation to IP-10. Comparison of miR-21 expression **(A)**, IP-10 mRNA level **(B)**, and IP-10 secretion **(C)** in THP-1 and THP-1 macrophages (THP-1-MA) cells. **(D)** Efficient overexpression of miR-21 did not reduce the secretion level of IP-10 in THP-1-MA cells stimulated by lipopolysaccharide (LPS). **(E)** Transfection with miR-21 inhibitors did not promote interferon-inducible protein 10 (IP-10) secretion in THP-1-MA cells stimulated by LPS. **(F)** Comparison of the relative expressions of ISG15 in THP-1 and THP-1-MA cells. **(G)** The suppression of ISG15 mRNA expression achieved by transfection of si-ISG15 to THP-1-MA cells. **(H,I)** Relative expression of IP-10 mRNA or IP-10 secretion after transfection of si-ISG15 itself, or with miR-21 mimics in the LPS-stimulated THP-1-MA cells.

Monocytes circulating in the blood stream continuously repopulate the macrophage population that resides within tissues (27). The vast majority of transcription factors are altered in the process of the differentiation monocyte to macrophage (28), which is an important source of the release of IP-10 (29). Therefore, we detected the alterations of miR-21 and IP-10 during the differentiation process from THP-1 to THP-1-MA cells. As illustrated in Figures 4A,B, the expression of miR-21 and IP-10 was remarkably more enhanced in THP-1-MA than in THP-1 cells (*P* = 0.002 and *P* = 0.037, respectively). We subsequently measured the IP-10 levels of unstimulated and stimulated THP-1 and THP-1-MA cells. The results showed a similar trend of increase in IP-10 secretion within the supernatant of THP-1-MA cells over that in THP-1 cells (*P* = 0.0008), especially after LPS stimulation (*P* = 0.0004; Figure 4C). We here asked why the increase of miR-21 during monocyte–macrophage differentiation was not accompanied by IP-10 inhibition, and we also wished to determine the potential of miR-21 to suppress IP-10 secretion in macrophages. In the results, we observed that miR-21 mimics or inhibitors did not play a significant role in IP-10 secretion of LPS-stimulated THP-1-MA cells (Figures 4D,E).

We then explored the potential mechanisms controlling the differential effects of miR-21 on IP-10 production between monocytes and macrophages. We speculated that there may have been some alteration of particular factors along the IP-10 signaling pathway altered in the THP-1-MA cells compared with the THP-1 cells, which may have attenuated the regulatory effect of miR-21 on IP-10 secretion (29). We found that IFN-stimulated gene 15 (*ISG15*), an IP-10 upstream molecule, underwent expression changes during the process of differentiation from THP-1 to THP-1-MA (30). ISG15 may trigger IP-10 expression by its function on NF-κB (31–33), which is a crucial transcription factor for IP-10 induction (34–37). This prompted us to investigate the role played by the enhanced level of ISG15 on IP-10 expression within THP-1-MA by comparison with the THP-1 cells (*P* = 0.010; Figure 4F). We propose that, through NF-kB activation, ISG15 regulates IP-10 at the transcription level. We found that the successful inhibition of ISG15 by siRNA (*P* = 0.017; Figure 4G) did significantly decrease both IP-10 mRNA expression (*P* = 0.039; Figure 4H) and protein production (*P* = 0.021; Figure 4I). This is different for miR-21. Our data indicated that post-transcriptional regulation carried out by miR-21 only acts on IP-10 protein production, but not IP-10 mRNA (Figures 3A–F). We found that the effect of miR-21 on IP-10 protein production becomes prominent after ISG-15 was silenced (*P* = 0.0009; Figure 4I). The results indicated that the transcriptional activation induced by enhanced ISG15 production is stronger than fine-tuning effect of post-transcriptional regulation mediated by miR-21. Therefore, overexpression of miR-21 cannot reduce the IP-10 protein production efficiently in THP-1-MA cells where ISG15 is highly expressed. Comparing to THP-1-MA cells, THP-1 cells express less ISG15 (Figure 4F). Nevertheless, suppression of ISG15 in THP-1 cells still led to reduced IP-10 mRNA expression (*P* = 0.014) and IP-10 protein production (*P* = 0.010) (Figures S3A,B in Supplementary Material). It is interesting to note that, since the basal level of ISG15 is reduced in THP-1 cells, we also observed that the silencing of ISG15 has a smaller effect on IP-10 production in comparison to the relative change in THP-1-MA cells (Figure S3C in Supplementary Material). These findings support the thesis that enhanced ISG15 levels in THP-1-MA facilitate IP-10 production and, therefore, overcome the inhibition of IP-10 by miR-21, potentially accounting for the differential effect of miR-21 to IP-10 expression in monocytes and macrophages.

## Discussion

HIV-1-associated immune dysregulation and activation is presumably established during the early stage of HIV-1 infection when a host of plasma cytokines and chemokines are rapidly developed (16, 38, 39). IP-10 is a pro-inflammatory chemokine expressed within a variety of cell types with pleiotropic function (40) and plays a pivotal role in regulating monocyte-mediated inflammatory responses as well as enhanced immune activation (41), thereby facilitating the disease progression of HIV (6, 10, 12, 13). In this study, we identified miR-21 as an important regulator of IP-10 in monocytes at post-transcriptional regulation level. For the THP-1-MA cells, this regulation is weakened as ISG15 is strengthened.

In this study, we first demonstrated that miR-21 regulates IP-10 secretion in monocytes. In our screening of six miRNA potential candidates, we observed that IP-10 in supernatant declined following the overexpression of miR-21 in LPS-stimulated THP-1 cells, and vice versa. Luciferase assay revealed that miR-21 directly targets IP-10 3′UTR through a predicted binding site (196–202). Previous studies performed on porcine kidney 15 cell lines and peritoneal macrophages from Sprague Dawley rats have showed that IP-10 was targeted by miR-21 (42, 43). Monocytes are one of the main sources of IP-10 (16), no study examining the post-transcriptional regulation of IP-10 in human monocytes has been reported to our knowledge. Therefore, using the THP-1 cell line, we found and report herein for the first time that miR-21 can regulate the expression of IP-10 in human monocytes. In HIV infection, it has been reported that increased circulating miR-21 in sera was able to discriminate HIV-infected from the HIV-uninfected groups (44) and correlated with soluble biomarkers of immune activation, including D-dimer and IL-6 (45). But whether miR-21 was altered in primary monocyte in HIV infection has not been reported. Our subsequent analyses of primary monocytes from HIV-infected patients showed the decreased expression of miR-21 and a trend of variation in miR-21 expression opposite to IP-10 levels in plasma. And transfection of miR-21 mimics has the tendency to decrease the LPS-stimulated IP-10 production by primary monocytes from HIV-infected patients. The result suggests that miR-21 may have the potential to regulate IP-10 production by monocyte in HIV infection. IP-10 promotes inflammation and activation, which associate with HIV disease progression and are difficult to be completely eliminated in HIV infection (2–4, 6, 10–12). We found that miR-21 could effectively inhibit the expression of IP-10 by monocytes, which is an important source of inflammation in HIV infection (46). Providing that the miRNA oligonucleotides (e.g., miR-122) have been previously employed in clinical control of infectious diseases (47), our results indicate the potential role of miR-21 as complementary therapeutic regimen for HIV infection by controlling the level of inflammation.

Although we have observed the importance of the role of miR-21 in regulating IP-10 at the post-transcriptional level of monocytes, this relationship was not observed in THP-1-MA cells. We detected the levels of expression of miR-21 and IP-10 during the differentiation process from monocyte to macrophage. We found both miR-21 and IP-10 were upregulated during the differentiation from THP-1 to THP-1-MA. This change in IP-10 expression may benefit the secretion of IP-10 and promote proinflammatory activation. However, it has not yet been satisfactorily explained why the effective overexpression of miR-21 did not reduce the generation of IP-10 in THP-1-MA cells in our study. We postulated that during the differentiation of monocyte to macrophage, some key genes regulating the expression of IP-10 altered and this weakens the regulation of miR-21 to IP-10. By exploring the signaling pathway and reviewing previous reports, we proposed an ubiquitin-like protein, ISG15 as essential in regulating the IP-10 expression and it exhibited higher levels during monocyte–macrophage differentiation and HIV infection (30, 48). Recent study revealed that ISG15 can induce the expression of IP-10 in *Candida albicans* infection (49). NF-κB is a well-known transcription factor for IP-10 gene activation (34–37). Previous studies showed that ISG15 enhances NF-κB activation by negatively regulates protein phosphatase 2Cβ (PP2Cβ) activity (31), which downregulated NF-κB activation by altering IKK activity (33). We postulated ISG15 may increase IP-10 production by the activation of NF-κB in THP-1-MA cells. Simmons et al. have described a connection between the plasma levels of IP-10 and ISG15 expression in HIV infection (16). According to our results, the process of cell differentiation from THP-1 to THP-1-MA is characterized by a conspicuous increase of ISG15 expression. The inhibition of ISG15 expression by siRNA triggers the recovery of miR-21 function to suppress IP-10 secretion in THP-1-MA cells. We proposed here that IP-10 is under the control of both ISG15 and miR-21. miR-21 directly targeting IP-10 at the post-transcriptional level while IP-10 is also regulated by ISG15 independently at the transcription level. The impact of ISG15 and miR-21 on IP-10 protein production is not equal. Our data indicated that enhanced ISG15-mediated transcriptional activation can overcome the post-transcriptional suppression of miR-21 in THP-1-MA cells. When ISG-15 was silenced, the effect of miR-21 on IP-10 production becomes prominent. The current study offers a comprehensive understanding of the regulation of IP-10 in HIV infection.

To conclude, we report the potential of miR-21 for regulating IP-10 expression in monocytes, whereas the elevated expression of ISG15 weakens the regulation of IP-10 by miR-21 in macrophages. Upregulated IP-10 in HIV infections is associated with high inflammation levels, rapid loss of CD4^+^ T cells, promoting infection by enabling viral reservoirs. Therefore, reducing IP-10 production is a promising and critical way of controlling HIV-associated inflammation and disease progression. Our results deepen the understanding of HIV pathogenesis and pave the way for inflammation intervention in HIV infection.

## Ethics Statement

The ethics approval was obtained from the First Hospital of China Medical University, and all the investigated participants were informed about the collection of blood samples, and provided written consent prior to enrolment in the study.

## Author Contributions

HS, Z-NZ, and XW conceived and designed the experiments; XW and L-LZ performed the experiments; XW analyzed the data; L-BY, Y-JF, Y-JJ, H-BD, and Z-XC contributed reagents/materials/analysis tools; XW and Z-NZ wrote the paper.

## Conflict of Interest Statement

The authors declare that the research was conducted in the absence of any commercial or financial relationships that could be construed as a potential conflict of interest.
