# Supplementary material for: Deregulated MicroRNA-21 Expression in Monocytes from HIV-Infected Patients Contributes to Elevated IP-10 Secretion in HIV Infection
Source: Front Immunol. 2017 Sep 11;8:1122. doi: 10.3389/fimmu.2017.01122 (PMC5601991; doi:10.3389/fimmu.2017.01122)
Supplement: Supplementary file 1 [file Table_1.DOCX]

Table S1. Mimic, inhibitor and primer Sequences for experiments.

| **Mimic** | **Sense Sequence ( 5`- 3`)** | **Antisense sense Sequence ( 5`- 3`)** |
| --- | --- | --- |
| miR-15 | UAGCAGCACAUAAUGGUUUGUG | CAAACCAUUAUGUGCUGCUAUU |
| miR-16 | UAGCAGCACGUAAAUAUUGGCG | CCAAUAUUUACGUGCUGCUAUU |
| miR-21 | UAGCUUAUCAGACUGAUGUUGA | AACAUCAGUCUGAUAAGCUAUU |
| miR-135 | UAUGGCUUUUUAUUCCUAUGUGA | ACAUAGGAAUAAAAAGCCAUAUU |
| miR-200c | UAAUACUGCCGGGUAAUGAUGGA | CAUCAUUACCCGGCAGUAUUAUU |
| miR-503 | UAGCAGCGGGAACAGUUCUGCAG | GCAGAACUGUUCCCGCUGCUAUU |
| NC | UUCUCCGAACGUGUCACGUTT | ACGUGACACGUUCGGAGAATT |
| **Inhibitor** | **Sequence ( 5`- 3`)** | - |
| miR-21 | UCAACAUCAGUCUGAUAAGCUA | - |
| NC | CAGUACUUUUGUGUAGUACAA | - |
| **Gene** | **Forward primer sequence ( 5`- 3`)** | **Reverse primer sequence ( 5`- 3`)** |
| IP-10 | GCCATTCTGATTTGCTGCCT | TGATGGCCTTCGATTCTGGA |
| ISG15 | CTCTGAGCATCCTGGTGAGGAA | AAGGTCAGCCAGAACAGGTCGT |
| GAPDH | ACATCGCTCAGACACCATG | TGTAGTTGAGGTCAATGAAGGG |
