# Supplementary figures and images for: Deregulated MicroRNA-21 Expression in Monocytes from HIV-Infected Patients Contributes to Elevated IP-10 Secretion in HIV Infection
Source: Front Immunol. 2017 Sep 11;8:1122. doi: 10.3389/fimmu.2017.01122 (PMC5601991; doi:10.3389/fimmu.2017.01122)

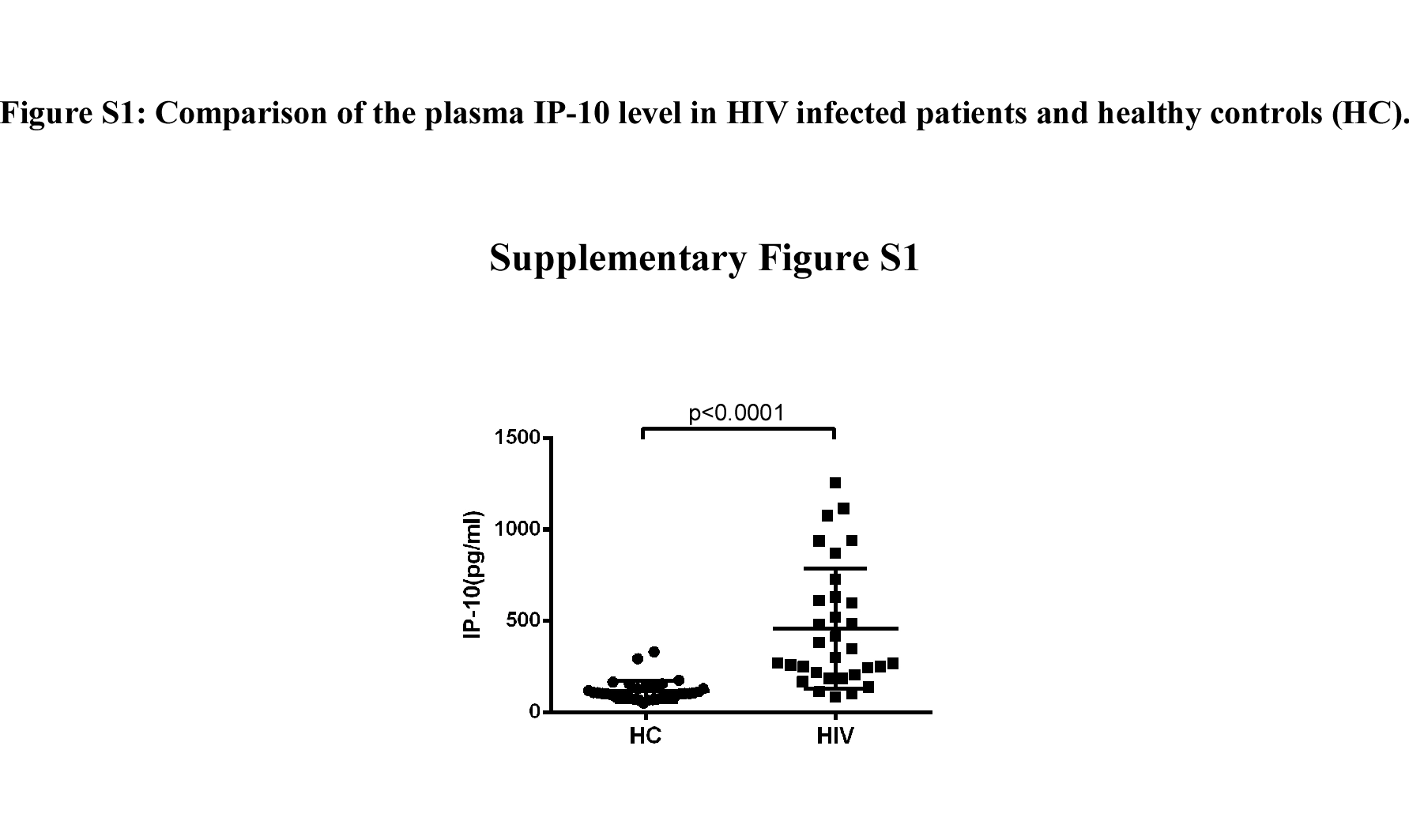

Supplement: Supplementary file 2 [file Image_1.TIF]

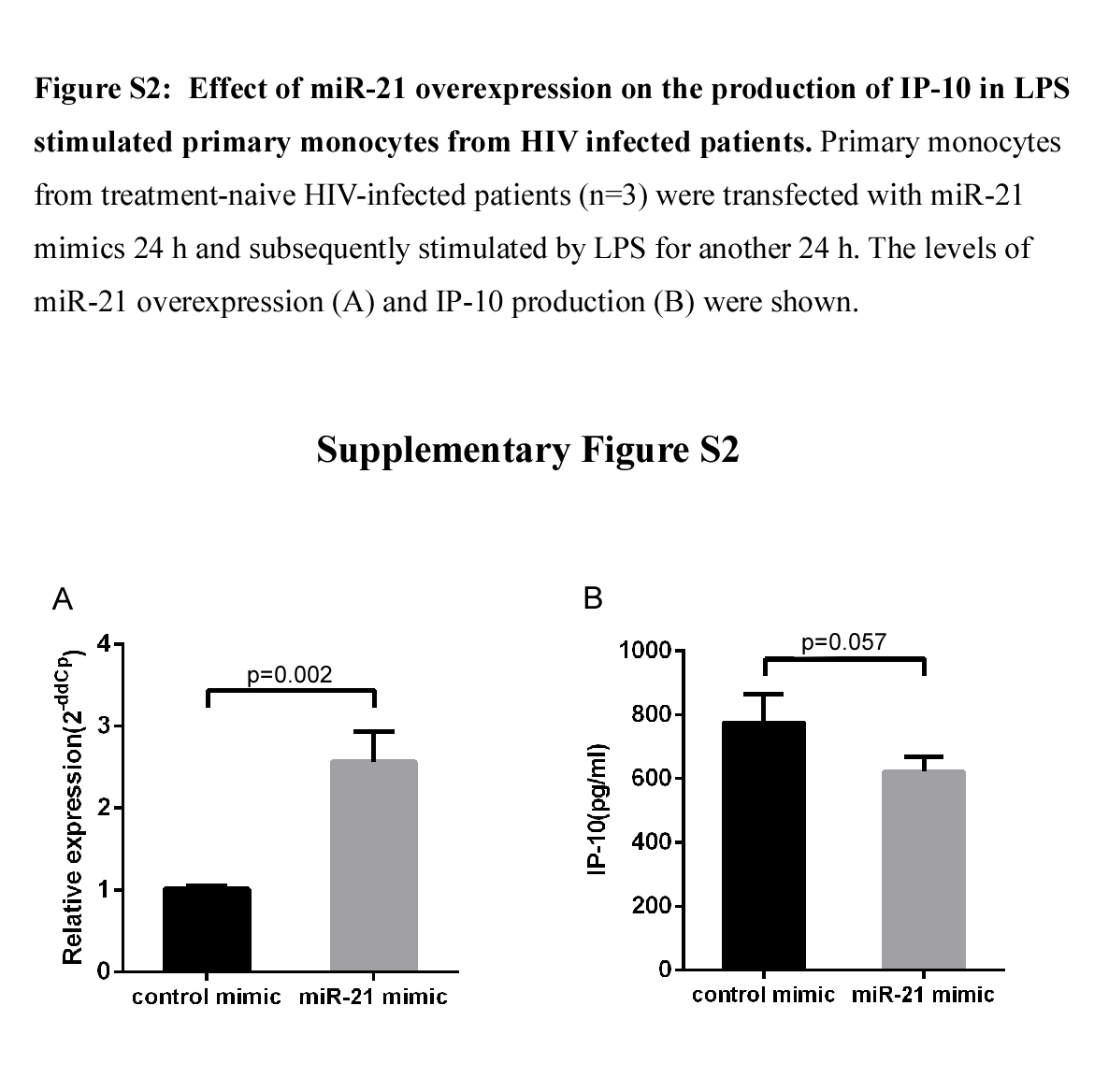

Supplement: Supplementary file 3 [file Image_2.TIF]

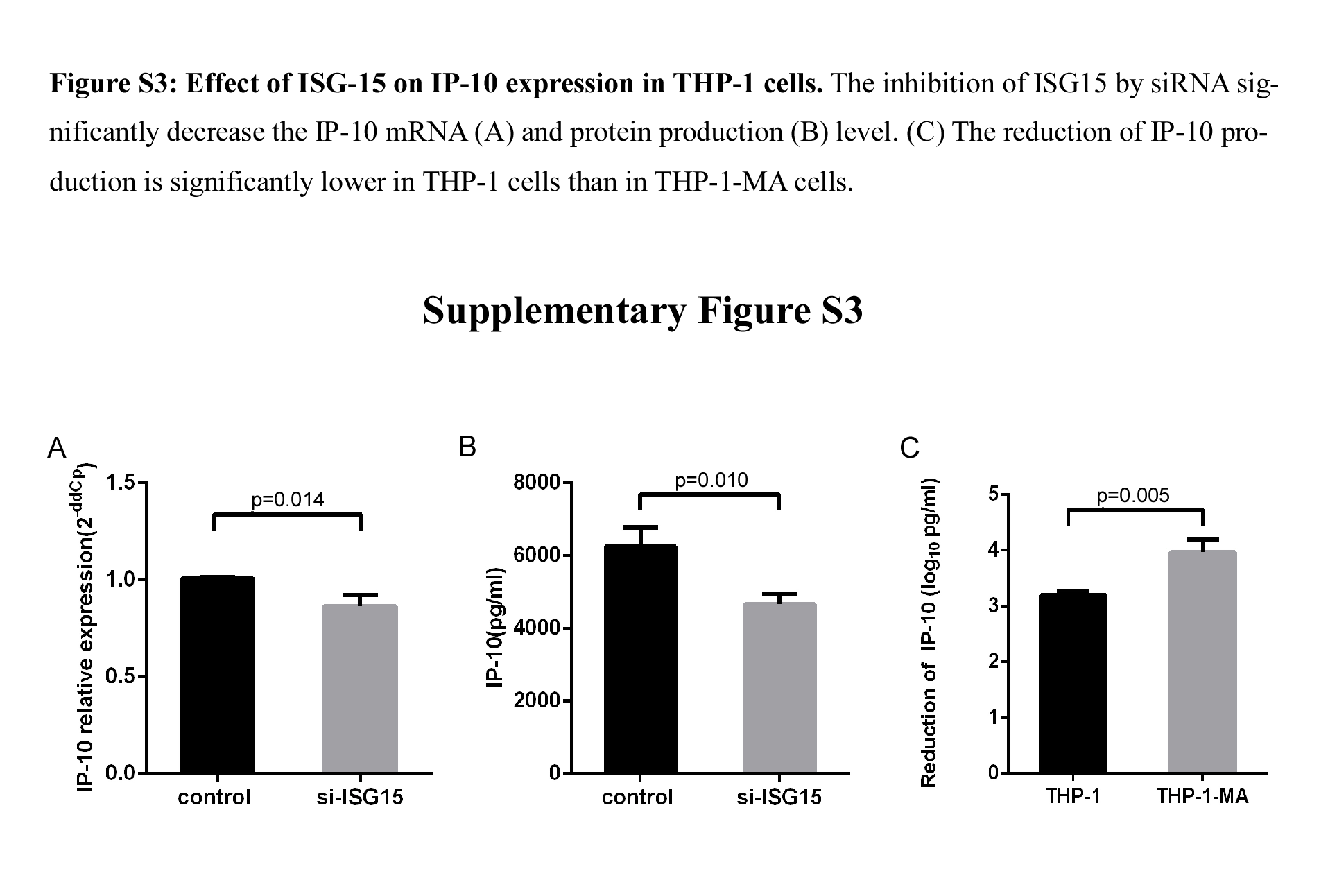

Supplement: Supplementary file 4 [file Image_3.TIF]
